# Editorial 2026

**DOI:** 10.1111/1755-0998.70093

**Published:** 2025-12-31

**Authors:** Benjamin Sibbett, Joanna Freeland, Joanna L. Kelley, Alana Alexander

**Affiliations:** ^1^ John Wiley & Sons Oxford UK; ^2^ Trent University Peterborough Canada; ^3^ University of California Santa Cruz Santa Cruz USA; ^4^ University of Otago Dunedin New Zealand

## 1 Journal Impact and Editorial Practices

Heading into 2026, *Molecular Ecology Resources* remains a leading publication for broad resources in the field of molecular ecology. This is supported by various citation‐based metrics, which continue to rank the journal highly in the related fields of Evolutionary Biology and Ecology. Our current Impact Factor (IF) of 5.5 places the journal 6th out of 53 in Evolutionary Biology and 21st out of 201 in Ecology in the Clarivate rankings. Other notable metrics additionally provide a positive picture for the journal, including the 5‐Year IF (8.0 compared to 7.8 in the previous year) and Scopus CiteScore (15.2 compared to 15.6 in the previous year). During 2025, *Molecular Ecology Resources* has responded to the continuously changing publishing landscape, details of which are described in this editorial; however, our mission in 2026 remains the same, which is to publish high‐quality resources that are of broad impact and pertinence to the community. To achieve this goal, we strive to offer a rigorous review process while supporting our authors with a fair and responsive experience.

## 2 Editorial Board

We wish to take this opportunity to express our enormous gratitude to Shawn Narum, whose tenure as Editor‐in‐Chief of *Molecular Ecology Resources* will end at the close of 2025. Since taking on the role in 2014, Dr. Narum has been instrumental in shaping the journal's direction, guiding it into a highly respected resource for the molecular ecology community. Over the past decade, Shawn has ensured that the journal keeps pace with rapid developments in the field, including advances in environmental DNA methodologies (Rodríguez‐Ezpeleta et al. 2021) and genome assembly (Whibley et al. 2021), thus enabling the journal to remain at the forefront of innovation. We are pleased that Shawn will continue to be part of the team as he takes on a new role as Senior Editor for *Molecular Ecology*. Ben Sibbett, current co‐Editor‐in‐Chief, has the privilege of leading the journal into 2026.

In support of Ben Sibbett, we are thrilled to share that Joanna L. Kelley (University of California Santa Cruz) and Alana Alexander (University of Otago) have accepted the newly created position of Senior Editor. Joanna and Alana transition to this role from their respective positions of News and Views Editor and Associate Editor. As Senior Editors, Joanna and Alana will have significant influence on the strategic direction of the journal and will advise the Editor‐in‐Chief on new initiatives and matters of journal scope. Further, Joanna and Alana will lead our efforts to develop and publish special issues in emerging topics of broad interest to our readers. Together with Joanna Freeland (Trent University), who continues as Reviews Editor in 2026, Joanna and Alana form the Senior Editorial Board of *Molecular Ecology Resources*.

*Molecular Ecology Resources* and *Molecular Ecology* acknowledge the importance of a strong social media presence. With this in mind, we are pleased to share that Angel G. Rivera‐Colón has been appointed Social Media Editor for both journals. Angel has previously been a key member of the journal's Junior Editor Board and will now spearhead our efforts to engage with the community and highlight the excellent research published in the journals. We congratulate Angel on this appointment.

In addition to changes to the Senior Editorial Board, we recognise that the peer review process is facing new and ongoing challenges, placing greater time demands on our Associate Editors. With year‐on‐year growth in scholarly content, it is unsurprising that it has become increasingly difficult to obtain expert assessment of submitted manuscripts. Furthermore, we realise that additional resource is required to ensure we remain vigilant against growing threats in the industry, such as the accelerating risks of fraudulent material in the form of paper mills and entirely AI‐generated manuscripts. To address these challenges, particularly maintaining robust and timely peer review, we will be pivoting to the use of professional editors at *Molecular Ecology Resources* from 2026. Professional editors joining the editorial board will be highly qualified, with PhDs in a relevant field and strong peer review experience and will work under the strategic direction of the Editor‐in‐Chief. While we acknowledge the need for this change, we take this opportunity to express our profound gratitude to our current Associate Editors who will step down at the end of 2025. By committing their valuable time and expertise to *Molecular Ecology Resources*, our departing Associate Editors have been instrumental to the success of the journal over many years, and we sincerely thank them for their service.

## 3 Open Access

A shift from the hybrid model (both open access (OA) and subscription content) to purely OA has been a serious topic of discussion at the journal since 2022 (Narum et al. 2023). In 2022, we acknowledged that an OA future looked highly likely for the journal, and since then, we have continued to see impressive growth in the proportion of content published OA versus subscription (proportion of content published OA: 2022 = 52%, 2023 = 60%, 2024 = 68%). Due to this, and the gathering pace of OA in ecology and evolutionary biology, we decided to make this transition in 2025. Consequently, all accepted articles submitted from August 27, 2025, will be published open access, ensuring the scientific content is immediately available to read and share without being confined behind a paywall.

The advantages of publishing open access for our authors are clear, with a study showing articles published open access have greater readership (3.5× views compared to subscription articles), are cited more (33% more citations compared to subscription articles), and receive greater Altmetric attention (almost 4× compared to subscription articles) (Wiley [White Paper], n.d.). With an increasing number of funder/institutional mandates for OA publishing, this transition ensures our authors remain compliant with such requirements and also allows authors to retain copyright over their valuable work. More broadly, global access to research findings drives forward innovation and collaboration, and we are pleased that this move aligns with our commitment to open science. We are excited to witness the above‐listed benefits come to fruition.

While thinking about OA, an important discussion point at *Molecular Ecology Resources* has been the article publication charge (APC), which can create a barrier to publishing for authors. We are especially mindful that available funds for APCs differ around the globe, thus disadvantaging authors from certain geographical locations. In this respect, we are pleased that our publisher, Wiley, has been finding ways of supporting authors while the industry adapts to OA. One important support pathway is the signing of new Transformational Agreements. These agreements migrate existing subscription spending to fund OA publishing, meaning authors covered by an agreement can often publish OA without direct cost. In addition, from January 2025, Wiley has been piloting a new programme that aims to support authors based in the Latin America region. This programme, termed the Open Access Pricing Power Parity Pilot, covers authors from 33 different countries and provides specific discounts based on Purchasing Power Index. Moreover, the continued partnership with Research4Life ensures eligible authors, including from across Africa, Asia and the Pacific Islands, are offered automatic waivers or discounts. Despite the growing support for APC payment, we acknowledge there is still work to be done, and we will continue to advocate for further directives and support mechanisms.

Finally, it is important to emphasize that the transition to OA will not impact the editorial standards of the journal. Manuscript evaluation, the peer review process, and our publishing ethics will remain independent and unchanged.

## 4 Highlights From 2025

Top resources published in *Molecular Ecology Resources* in the last year include key contributions across multiple subject areas. Articles may receive extra attention through publication as a ‘From the Cover’ article that is intended to be published in each issue along with a Perspective article. These articles were coordinated by News & Views Editor, Joanna Kelley. In the last year, articles on the cover of *Molecular Ecology Resources* focused on multiple new resources for the community including: tools that identify misassemblies and annotate sequences of interest (Klumpy; Madrigal et al. 2025) as well as repeats and putative centromeres (RepeatOBserver; Elphinstone et al. 2025), advances in eDNA approaches to estimate species abundance using segregating sites (Ai et al. 2025), an evaluation of the performance of genomic offset approaches (Lind and Lotterhos 2025), a strategy for metagenomics that uses unassembled reads from low‐coverage genomes and available reference genomes to construct genomic reference databases for biodiversity monitoring (Callens et al. 2025), imputing missing genotypes using self‐organising maps (Mora‐Márquez et al. 2025), and a method for obtaining historical DNA from museum samples (Holmquist et al. 2025). Editors also nominated additional high‐impact articles published in 2025 to be highlighted with perspectives. Those articles included a method for increasing precision of effective population size estimates (Waples et al. 2025) and new tools to estimate diversity using microsatellites from eDNA (Liggan et al. 2025).

Invited reviews continue to make important contributions by summarising up‐to‐date information and identifying knowledge gaps on a wide range of topics. In 2025 we published an Invited Review that provided an overview of different methods for calculating aging in natural populations based on epigenetic data (Newediuk et al. 2025). The ‘Mini Review’ category continued to provide a useful option for topics or emerging methodologies that merit a more concise summary, and this year we published Mini Reviews on genomic approaches for samples preserved in formalin (Holleley and Hahn 2025), the potential for genome skimming as a tool for generating data from environmental DNA (Lu et al. 2025), and rapid DNA/eDNA‐Based ID tools aimed at improving Chondrichthyan monitoring and management (Alvarenga et al. 2025). Additionally, a review of CRISPR‐Cas technologies in ecological studies is in press (Plewnia et al. 2026). The workload involved with all types of reviews and syntheses is substantial and we are very grateful to the authors that have provided informative summaries of topics that increasingly incorporate complex molecular data and analyses. Suggestions for review topics are always welcome; please contact the reviews editor (joannafreeland@trentu.ca) if you have ideas for future reviews.

*Molecular Ecology Resources* also publishes special issues, aimed to showcase emerging and topical research areas in the field. In 2025, the journal published two special issues. The first, titled ‘skúkum tílixam: Uniting to Support Indigenous Contributions to Molecular Ecology’ (Volume 25, Issue 2) provides an impressive collection of papers that highlight and acknowledge the trailblazing research contributions of Indigenous scientists and emphasise the value of Indigenous Research Methodologies (Ramos et al. 2025). The second special issue of 2025, ‘Advancing Species Conservation and Management Through Omics Tools’ (Volume 25, Issue 5) compiled 34 papers demonstrating the uses and applications of omics tools for management and conservation strategies across the tree of life (von der Heyden et al. 2025).

## 5 Thank You

We thank the large number of individuals who contributed to the journal in 2025. The following list contains people who have supported the journal and its community by reviewing articles for the journal between October 1, 2024 and September 30, 2025. Please note this list only contains names of reviewers who reviewed for the journal in our former ScholarOne system and does not include individuals who have provided reviews in our new Research Exchange platform, whom we hope to be able to acknowledge in next year's editorial.

Akita, Shingo

Allard, john

Alvarez Costes, Sebastian

Anderson, Eric

Andersson, Bea

Andres, Kara

Anslan, Sten

Antognazza, Caterina

Antonino, Jose Dijair

Arvestad, Lars

Barkan, Roy

Barteri, Fabio

Bayer, Philipp

Benham, Phred

Bennington, Stephanie

Bian, Chao

Bickham, John

Blackman, Heath

Bohmann, Kristine

Bolaj, Ayo

Bombarely, Aureliano

Borges, Luisa

Bourlat, Sarah

Bovo, Samuele

Brandão‐Dias, Pedro

Brandvain, Yaniv

Brejon Lamartinière, Etienne

Budd, Alyssa

Campagna, Leonardo

Campelo, Felipe

Caro‐Quintero, Alejandro

Carrera‐Martínez, Roberto

Carugati, Laura

Castellanos‐Labarcena, Jessica

Cecchetto, Matteo

Chafin, Tyler

Chang, Xianmin

Chang, Ying

Chatzinotas, Antonis

Chen, Stephanie

Chen, Zhong‐Hua

Chiappa, Giacomo

Cooke, Ira

Costantini, Federica

Crochet, Pierre‐Andre

Cuff, Jordan

curik, Ino

Curto, Manuel

da Silva, Jessica

de Brito, Reinaldo

de Carvalho, Clarissa

de Flamingh, Alida

De Sanctis, Bianca

Delomas, Thomas

Deng, Yanming

Deutsch, Kaitlin

Díaz‐Jaimes, Pindaro

Dierckxsens, Nicolas

Dietrich, Christopher

Ding, Shou Wei

Duan, Jun

Dunshea, Glenn

Dunthorn, Micah

Eisele, Marius

Ekrem, Torbjørn

Elkrewi, Marwan

Elliott, Tyler

Ellis, Vincenzo

Elphinstone, Cassandra

Ely, Taylor

Encinas‐Viso, Francisco

Eriksson, Charlotte

Escalona, Merly

Fagernäs, Zandra

Fan, Longjiang

Fan, Qiang

Fathi, Mohammad Reza

Fauver, Joseph

Fedosov, Alexander

Ferdosi, Mohammad

Fernández, Sara

Fieberg, John

Fiumera, Anthony

Flanagan, Sarah

Fontaine, Albin

Furneaux, Brendan

Galen, Spencer

Garcia‐Erill, Genís

Gerber, Livia

Giachello, Simone

Gill, Mandev

Girard, Elsa B.

Girard, Simon

Goesser, Fabian

Gomez‐Acata, Selene

Gossmann, Toni

Goulding, Tricia

Green, Eleanor

Grozinger, Christina

Grzebelus, Dariusz

Guhlin, Joseph

Guo, Fei

Guo, Zixiao

Hablützel, Pascal

Hao, Yan

Harder, Avril

Hardy, Olivier

Harrison, Hugo

Hayward, Kristen

He, Xiaoping

Hedtke, Shannon

Herbold, Craig

Hernandez‐Triana, Luis

Hiillos, Anna‐Lotta

Höglund, Jacob

Holsinger, Kent

Horvath, Steve

Huang, Kaichi

Huang, Kang

Huber, Gwendolyn

Isabel Vester, Kristen

Janes, Jasmine

Jansen, Lara

Jarman, Simon

Jiang, Yu

Jo, Toshiaki

Johnson, Kevin

Johnson, Mark

Juhel, Jean‐Baptiste

Kane, Nolan

Kartzinel, Tyler R.

Kellner, Kenneth

Kikuchi, Kiyoshi

Kireta, Dona

Kizis, Dimosthenis

Kjesbu, Olav

Klymus, Katy

Knott, Karelyn

Kocot, Kevin

Köninger, Julia

Krabbenhoft, Trevor

Kuelheim, Carsten

Kulkarni, Siddarth

Lan, Tianming

Lara, Enrique

Laroche, Olivier

Latch, Emily

Le Gac, Mickael

Lee, Cheng‐Ruei

Leempoel, Kevin

Lentendu, Guillaume

Lepais, Oliver

Leugger, Flurin

Levis, Nicholas

Lewanski, Alexander

Li, Chenhong

Liao, Ben‐Yang

Lin, Yuling

Lind, Brandon

Lindner, Melanie

Littlefair, Joanne

Liu, Fang

Liu, Jian‐Feng

Liu, Shanlin

Liu, Xingyue

Lloyd‐Jones, Luke

Lockwood, Julie

López de Heredia, Unai

Lopez, Mark Louie

Lovell, John

Lynggaard, Christina

Ma, Wen‐Juan

Maiello, Giulia

Mairal, Mario

Malenfant, René

Mao, Kangshan

Marcellino, Jose

Marin, Mario Alejandro

Martin, Claudia

Martin, Michael

Martínez Portela, Paulino

Maruki, Takahiro

McDevitt, Allan

McDonald, Ian

McKernan, Kevin

McTaggart, Alistair

Meier, Rudolf

Meiklejohn, Kelly A.

Melo‐Ferreira, José

Mendez Monroy, Paul Erick

Mercier, Céline

Merten Cruz, Marcelo

Meyermans, Roel

Mikhailov, Ivan

Mikryukov, Vladimir

Minamoto, Toshifumi

Monlong, Jean

Mooney, Jazlyn

Morales, Ariadna

Munguia‐Vega, Adrian

Munian, Kaviarasu

Nakajima, Souta

Nanni, Loris

Newton, Joshua

Nijland, Reindert

Nilsson, Henrik

Novotny, Vojtech

Octavia, Sophie

Oggenfuss, Ursula

Oleksyk, Taras

Olofsson, Jill

Ottewell, Kym

Panahi, Bahman

Pang, Rui

Paradis, Emmanuel

Paris, Margot

Parle‐McDermott, Anne

Patterson, Gilia

Pearman, William

Pekarik, Ladislav

Peng, Bo

Penna, Anna

Perl, Bina

Perry, George

Pezzi, Pedro

Pierella Karlusich, Juan José

Pinol, Josep

Pioltelli, Emiliano

Plewnia, Amadeus

Pohjoismäki, Jaakko

Pont, Didier

Porth, Ilga

Price, Ben

Prondzinsky, Paula

Ramos, Seafha

Rawlence, Nic

Recknagel, Hans

Richmond, Robert

Ringbauer, Harald

Robinson, John

Robledo‐Ruiz, Diana

Rollins, Lee

Rondeau, Eric

Roossinck, Marilyn

Rossel, Sven

Rout, Ajaya Kumar

Saitou, Marie

Sales, Naiara

Sanchez, Daniel

Sandkam, Benjamin

Şapcı, Ali Osman Berk

Schöneberg, Yannis

Schroeder, Anna

Scott, Peter

Seaborn, Travis

Seashols‐Williams, Sarah

Seersholm, Frederik

Sepulveda, Adam J.

Sessegolo, Camille

Seto, Kensuke

Seymour, Mathew

Shafer, Aaron

Shu, Kai

Shum, peter

Silva‐Arias, Gustavo

Smith, Martin

Snyder, Elise

Solari, Katherine

Soto, Álvaro

Sousa, Vitor C.

Speller, Camilla

Srivathsan, Amrita

Steel, Debbie

Stelbrink, Björn

Stoeck, Thorsten

Stoof‐Leichsenring, Kathleen

Stothart, Mason

Suchan, Tomasz

Tangili, Marianthi

Tao, Xiang

Taylor, Katherine

Tedersoo, Leho

Thalmann, Olaf

Thompson, Anne

Tibone, Maddalena

Trujillo‐González, Alejandro

Turon, Xavier

Twyford, Alex

van der Heyde, Mieke

van der Reis, Aimee

Vaughn, Andrew

Vaulot, Daniel

Velsko, Irina

Vences, Miguel

Vernesi, Cristiano

Vieites, David

Vierstraete, Andy

Villoutreix, Romain

Vogler, Alfried

VV, Robin

Wambugu, Peterson

Wang, Kun

Wang, Qinhu

Wang, Shuping

Wang, Wen

Wang, Xiaotong

Wang, Youji

Waples, Robin

Watanabe, Kozo

Weber, D. Nick

Weber, Sven

Wells, Caitlin

Weng, Yi‐Ming

Westbury, Michael

Wilcox, Taylor

Willis, Stuart

Worth, James Raymond Peter

Wray, Amy

Wright, Charlotte J.

Wright, Sterling

Wu, Luhan

Xia, Pu

Xiong, Wen

Yadav, Shweta

Yasuda, Nina

Yen, Eugenie

Yi, Xueling

Zaharias, Paul

Zhang, Jianzhi

Zheng, Huaiping

Zhou, Qing‐Song

Zhou, Xuming

Zhu, Ying

Zoclanclounon, Yedomon Ange Bovys

## Conflicts of Interest

The authors declare no conflicts of interest.

## Data Availability

The authors have nothing to report.
